# Nogo receptor complex expression dynamics in the inflammatory foci of central nervous system experimental autoimmune demyelination

**DOI:** 10.1186/s12974-016-0730-4

**Published:** 2016-10-11

**Authors:** Paschalis Theotokis, Olga Touloumi, Roza Lagoudaki, Evangelia Nousiopoulou, Evangelia Kesidou, Spyridon Siafis, Theodoros Tselios, Athanasios Lourbopoulos, Dimitrios Karacostas, Nikolaos Grigoriadis, Constantina Simeonidou

**Affiliations:** 1 B’ Department of Neurology, Laboratory of Experimental Neurology and Neuroimmunology, AHEPA University Hospital, Aristotle University of Thessaloniki, Stilponos Kiriakides str. 1, 546 36 Thessaloniki, Central Macedonia Greece; 2 Department of Chemistry, University of Patras, Rion, 265 04 Patras, Greece; 3 Institute for Stroke and Dementia Research (ISD), Feodor-Lynen-Strasse 17, 81377 Munich, Germany; 4 Department of Experimental Physiology, Faculty of Medicine, Aristotle University of Thessaloniki, 546 36 Thessaloniki, Central Macedonia Greece

**Keywords:** Nogo receptor complex, NgR, LINGO-1, p75, TROY, Experimental autoimmune encephalomyelitis, Neuroinflammation, Macrophages

## Abstract

**Background:**

Nogo-A and its putative receptor NgR are considered to be among the inhibitors of axonal regeneration in the CNS. However, few studies so far have addressed the issue of local NgR complex multilateral localization within inflammation in an MS mouse model of autoimmune demyelination.

**Methods:**

Chronic experimental autoimmune encephalomyelitis (EAE) was induced in C57BL/6 mice. Analyses were performed on acute (days 18–22) and chronic (day 50) time points and compared to controls. The temporal and spatial expression of the Nogo receptor complex (NgR and coreceptors) was studied at the spinal cord using epifluorescent and confocal microscopy or real-time PCR. Data are expressed as cells/mm^2^, as mean % ± SEM, or as arbitrary units of integrated density.

**Results:**

Animals developed a moderate to severe EAE without mortality, followed by a progressive, chronic clinical course. NgR complex spatial expression varied during the main time points of EAE. NgR with coreceptors LINGO-1 and TROY was increased in the spinal cord in the acute phase whereas LINGO-1 and p75 signal seemed to be dominant in the chronic phase, respectively. NgR was detected on gray matter NeuN^+^ neurons of the spinal cord, within the white matter inflammatory foci (14.2 ± 4.3 % NgR^+^ inflammatory cells), and found to be colocalized with GAP-43^+^ axonal growth cones while no β-TubIII^+^, SMI-32^+^, or APP^+^ axons were found as NgR^+^. Among the NgR^+^ inflammatory cells, 75.6 ± 9.0 % were microglial/macrophages (lectin^+^), 49.6 ± 14.2 % expressed CD68 (phagocytic ED1^+^ cells), and no cells were Mac-3^+^. Of these macrophages/monocytes, only Arginase-1^+^/NgR^+^ but not iNOS^+^/NgR^+^ were present in lesions both in acute and chronic phases.

**Conclusions:**

Our data describe in detail the expression of the Nogo receptor complex within the autoimmune inflammatory foci and suggest a possible immune action for NgR apart from the established inhibitory one on axonal growth. Its expression by inflammatory macrophages/monocytes could signify a possible role of these cells on axonal guidance and clearance of the lesioned area during inflammatory demyelination.

**Electronic supplementary material:**

The online version of this article (doi:10.1186/s12974-016-0730-4) contains supplementary material, which is available to authorized users.

## Background

Numerous studies indicate the dynamic and high potential role of neurite outgrowth inhibitor Nogo-A to inhibit, guide, and modulate the injured and demyelinating tissue in various models of disease. These characteristics are dependent on the presence of Nogo-66 receptor (NgR) [1] as well as the subcellular coreceptor components p75 [2], TROY [3], and adaptor molecule LINGO-1 [4], comprising the receptor complex that mediates myelin’s inhibitory action. The latest finding as a component of this complex is AMIGO3, substituting LINGO-1 under specific circumstances [5].

NgR is expressed by various glial and neuronal cell bodies or axons except oligodendrocytes, which express its ligand Nogo-A [6, 7]. This specific ligand-receptor localization has been accounted for a neurite outgrowth inhibitory mechanism, exerting a growth cone collapse effect in response to at least two more myelin proteins, namely MAG [8] and OMgp [9]. The 473-amino acid NgR protein is a glycosylphosphatidylinositol (GPI)-linked molecule linked to an eight-leucine-rich-repeat (LRR) domain by an LRR carboxy-terminal (LRRCT) domain. It can be found in three isoforms: NgR1, NgR2, and NgR3 [10], with NgR1 being the most common and best correlated to Nogo-A [11]. The other three molecules of the NgR complex, namely p75, TROY, and LINGO-1, are membrane coreceptors that regulate the downstream effects of NgR depending on their interactions [12, 13].

The value of the Nogo-NgR pathway for control of axonal regeneration is verified by the development of Nogo-A and LINGO-1 monoclonal antibodies for clinical trials [14, 15]. Despite the fact that NgR is key to the pathway activation, only few studies analyzing knockout mice in experimental autoimmune encephalomyelitis (EAE) [16, 17] and few small interference RNA (siRNA) experiments have been carried out in demyelination of optic nerve tracts [18, 19], rendering its precise function still enigmatic. In vitro experiments by Takahashi and colleagues [20] and later further progressed by David and colleagues [21, 22] suggest that the presence of NgR in macrophages—apart from neurons or glia—could support the clearance of debris and could confine the injury away from the normal-appearing white matter tissue. However, the question of whether the NgR complex positively or negatively regulates the inflammation spreading in a central nervous system (CNS)-based autoimmune disease remains largely unknown so far.

The purpose of this study was to describe the spatiotemporal kinetics of NgR within the inflammatory sites of experimental autoimmune demyelination, in order to understand its roles in the inflammatory milieu. We focused on both NgR and coreceptor molecules LINGO-1, p75, and TROY, in an attempt to characterize the Nogo-A/NgR pathway in the autoimmune inflammatory foci. Based on our present and previous published data [23], we propose that NgR may have an additional role in the disease progression—besides the attributed axonal inhibition-, namely confining the inflammatory reaction and/or the sprouting of axons in EAE lesions.

## Methods

### Animal handling

Female C57BL/6 mice (*n* = 40), 8–10 weeks old, were purchased from the Hellenic Pasteur Institute (Athens, Greece) and housed in the pathogen-free animal facility of the B’ Neurology Department, AHEPA University Hospital, Thessaloniki, Greece. Animals were fed a normal diet and given ad libitum water without antibiotics. All experimental procedures were conducted according to the institutional guidelines, in compliance with the Greek Regulations and the European Communities Council Directive of November 24, 1986 (86/609/EEC). Experimentation received approval from the Veterinary Medicines Directorate (license number 177867/1510).

### EAE induction and clinical evaluation

Chronic EAE was induced in animals (*n* = 30), with subcutaneous injection of 300 μg of myelin oligodendrocyte glycoprotein 35–55 peptide (MOG_35–55_) as previously described [23, 24]. Animals inoculated with complete Freund’s adjuvant (CFA, supplemented with 4 % mycobacterium tuberculosis) only did not develop lesions in the CNS and served as controls (*n* = 10). All animals were examined daily using a 6-grade scale: 0, asymptomatic; 1, partial loss of tail tonicity; 2, flaccid tail paralysis; 3, difficulty to roll over from a supine position; 4, hind limb paralysis; 5, forelimb paresis; and 6, death due to EAE.

The following indexes were calculated based on their daily scores: mean maximal score (MMS), area under the curve (AUC), day of disease onset (dDO), clinical score at day 50 (d50 score), and demyelination extent in the spinal cord.

### Tissue collection

Animals were humanely euthanized and prepared accordingly for neuropathology and further processing. Animals for immunohistochemistry were transcardially perfused using phosphate buffer saline (PBS), followed by ice-cold 4 % paraformaldehyde in PBS (4 % PFA) for 5 min. The brain and spinal cords were removed, postfixed in the corresponding fixation solution for 16–20 h at 4 °C, and routinely processed for paraffin sectioning at 6 μm. Animals for real-time PCR were euthanatized, and their brains and spinal cords were removed, snap frozen, and stored at −80 °C until further use.

### Histological stainings, immunohistochemistry, and immunofluorescence

Six-micrometer-thick paraffin coronal spinal cord sections were stained with routinely used immunohistochemical methods: Luxol fast blue (LFB) counterstained with nuclear fast red for demyelination evaluation and Bielschowsky silver impregnation counterstained with hematoxylin for pervasive infiltratory burden and axonal loss, as previously described [25–27]. Lectin and DAB-based anti-Iba-1 (rabbit, WAKO) protocols were used for microglia evaluation, as previously described [27].

Localization and neuropathological study of NgR was performed with double immunofluorescence (dIF) also on 6-μm coronal sections. Briefly, following hydration and incubation of the sections with 10 % fetal bovine serum (FBS) for 30 min, primary antibodies were applied overnight. Neuronal and glial components were evaluated for the presence of NgR with a combination of primary antibodies, anti-NgR (rabbit, Santa Cruz) with anti-NeuN (mouse, Millipore) for neurons, anti-GFAP (mouse, DAKO) for astrocytes, anti-Nogo-A (goat, Santa Cruz) for mature oligodendrocytes, anti-Mac-3 (rat, BD Biosciences) for detection of peritoneal/tissue macrophages and dendritic cells, and anti-ED1 (mouse, Serotec) for detection of macrosialin (macrophages; human CD68), while anti-Arginase-1 (goat, Santa Cruz) and anti-iNOS (mouse, Santa Cruz) were used to distinguish M2 microglia from the M1 phenotype [28].

Additionally, axonal epitopes were designated with the following: anti-β-TubIII (mouse, BD Biosciences) and non-phosphorylated neurofilament H anti-SMI-32 (mouse, Calbiochem) were used for detection of physiological and pathological neuronal axons, and anti-β-amyloid precursor protein (APP) (mouse, Millipore) and anti-GAP-43 (mouse, Sigma) were used for axonal focal degeneracy and regeneration, respectively. Coreceptors LINGO-1 (rabbit, Abcam ab23631, 1:300), TROY (goat, Santa Cruz 13711, 1:100), and p75 (mouse, Abcam ab8877, 1:400) were also accessed and combined accordingly. For specificity and quality control of the coreceptor antibodies, a preabsorption protocol previously described [29] and the use of a second antibody recognizing a different epitope were applied upon availability and whenever applicable (see Additional file 1: Figure S1). Briefly, the peptide for each preabsorption assay was mixed with the antibody in a 10× molecular ratio and was incubated under gentle agitation at room temperature for 30 min. The mixture was then used in place of the primary antibody for the rest of the IF protocol.

Depending on the primary antibodies used, the following secondary fluorescent antibodies were used at the spectrum of green (488, Biotium; Alexa Fluor) and red (555-568, Biotium; Alexa Fluor) for the rest of the markers. Sections were mounted with 4′,6-diamidino-2-phenylindole (DAPI) (Biotium).

### Neuropathology evaluation

Pathologic examination of demyelination, axonopathy, inflammation, and microglia/macrophages was performed under a Zeiss Axioplan 2 light microscope by two, blinded to the experimental groups, investigators. Photos were captured with the aid of a CCD camera (Nikon), and analysis of the designated areas was performed with ImageJ software.

Demyelination was evaluated on approximately 15 Luxol fast blue-stained sections. Demyelinated areas and the total area of white matter were carefully circumscribed and expressed as a percentage (demyelinated/total wm area), as previously described [23]. Axonal loss was assessed on approximately ten randomly selected silver-stained spinal cord sections within 150 × 150-μm areas from the three major spinal cord white matter columns (ventral, lateral, and dorsal), spaced at least 60 μm apart, and graded as 0 = normal/even silver stain throughout the white matter compared to unimmunized mice; 1 = small spurious areas in the white matter that lack silver stain; 2 = small, but frequent, areas in the white matter that lack silver stain; and 3 = extensive loss of silver stain throughout the white matter, as previously described [25]. Typical images of scores 0–3 are presented in Additional file 1: Figure S1.

For the evaluation of inflammation, hematoxylin-stained fields of the same sections were examined for each animal, using a prefrontal microscope grid. Microglia/macrophagic populations were assessed in respective white matter areas with ionized calcium-binding adaptor molecule-1 (Iba-1) staining. The number of perivascular infiltrates and microglia/macrophages were counted and presented as cells/mm^2^.

### NgR profile assessment

Spatial analysis for NgR protein was performed in the center of six to eight inflammatory foci per spinal cord per animal, spaced at least 50 μm apart. Sections were studied using confocal microscopy (Nikon C1-Eclipse TE-2000U) under ×60 optical fields. Briefly, images were captured, within a predefined 305 × 305-μm area, and the NgR-positive signal was evaluated for levels of coexpression with miscellaneous markers. GAP-43 and APP signal was measured as an integrated density (IntDen) as previously described [23], using the ImageJ software. Colocalization percentages were also estimated, wherever applicable.

### NgR coreceptor real-time PCR

Molecular analysis of coreceptors LINGO-1, TROY, and p75 was performed with real-time PCR in spinal cord extracts based on a protocol previously described [23]. Briefly, total RNAs were extracted from spinal cord tissues using Trizol reagent (Invitrogen); RNA was reverse transcribed to complementary DNA (cDNA) with iScript cDNA Synthesis Kit (BioRad), and real-time PCR was performed using BioRad IQ5 ICycler Multicolor Detection System. The relative gene expression was normalized to β-actin which served as an internal control. Additionally, GAPDH was used as a second, quality control, house-keeping gene. Primer sequences for SYBR Green probes of target genes are as follows: LINGO-1 (Lingo1): forward 5′ CATCAGGTGAGCGAGAGGAT 3′ and reverse 5′ CGTCCTGGTTGAGTGTCTTG 3′ giving rise to a 267-bp product; p75 (Ngfr): forward 5′ CTGCTGCTTCTAGGGGTGTC 3′ and reverse 5′ ACACAGGGAGCGGACATACT 3′ giving rise to a 248-bp product; TROY (Tnfrsf19): forward 5′ AGATTGCAGGCAGCAGGA 3′ and reverse 5′ TCCGCACATGGCTTACACTT 3′ giving rise to a 186-bp product; β-actin (Actb): forward 5′ TTGTAACCAACTGGGACGATATGC 3′ and reverse 5′ GATCTTGATCTTCATGATGCTAGG 3′ giving rise to a 139-bp product; and GAPDH (Gapdh): forward 5′ GGATGCAGGGATGATGTTCT 3′ and reverse 5′ AAGGGCTCATGACCACAGTC 3′ giving rise to a 116-bp product. The results were analyzed using ΔΔCt method.

### Statistical analysis

All data are given as mean ± standard error of the mean (SEM). For statistical analysis, the SPSS Statistics 18 package was used. Student’s *t* test and one-way analysis of variance (ANOVA) with Dunnett’s and Bonferroni post hoc tests were used for comparisons of two or more groups, respectively. Non-parametric data were compared using Mann-Whitney *U* test. Semi-quantitative data were analyzed using the Pearson *χ* ^2^ test or Fisher’s exact test, where appropriate, and the ordinal data were displayed as bar graphs. Two-tailed values of *p* < 0.05 were considered statistically significant for all tests.

## Results

### Clinical course and inflammatory pathology

**Fig. 1:**
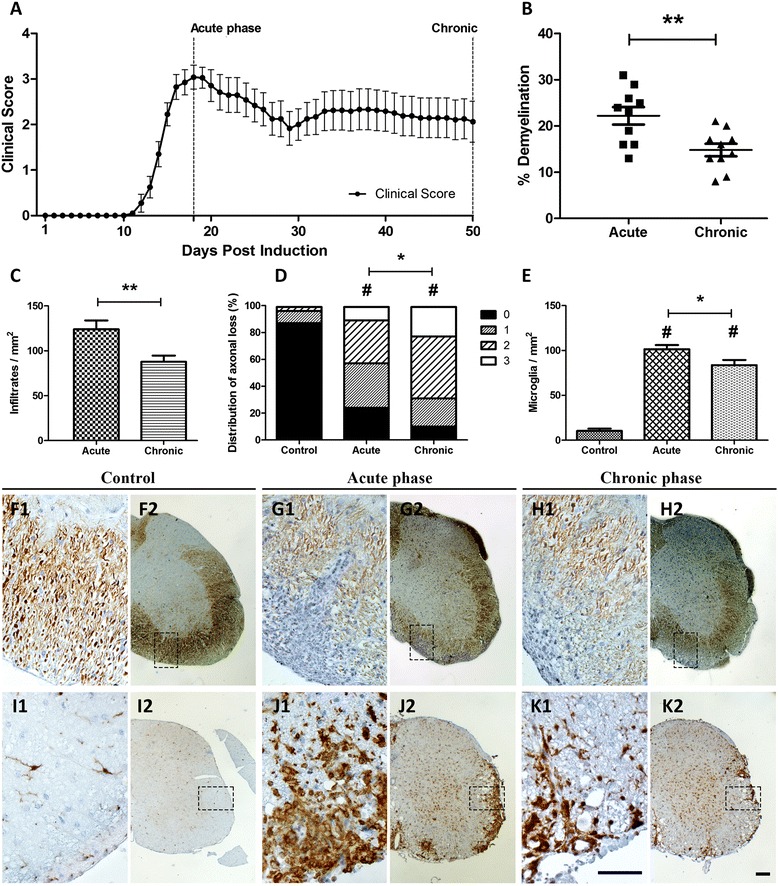
Clinical course, demyelination, infiltratory, axonal, and microglial status in the spinal cord of EAE animals. **A** Graphic depicts the EAE course and the two main time points analyzed. **B** % demyelination as resulted by LFB staining. On the chronic phase, demyelination was significantly lower compared to the acute phase (*p* < 0.01). Infiltratory burden (**C**) expressed as cells/mm^2^ and axonal loss (**D**) measured semi-quantitatively (0 = normal/even silver stain throughout the white matter; 1 = small spurious areas in the white matter that lack silver stain; 2 = small, but frequent, areas in the white matter that lack silver stain; and 3 = extensive loss of silver stain throughout the white matter) were computed from the same sections of a modified Bielschowsky protocol counterstained with hematoxylin (panels **F2**, **G2**, **H2**, and their corresponding field magnifications **F1**, **G1**, and **H1**). Perivascular infiltrations were significantly higher in the acute phase compared to chronic (*p* < 0.01) while axonal loss was found higher in the chronic phase compared to acute (*p* < 0.05). Panels **I2**, **J2**, and **K2** show the adjacent sections of the spinal cord stained for microglia/macrophages (Iba-1^+^; panels **I1**, **J1**, and **K1** show corresponding field magnifications). Microglia (**E**, cells/mm2) differed significantly between control and EAE animals (*p* < 0.001 and *p* < 0.05 for acute and chronic phases, respectively). *Error bars* indicate the standard statistical error of the mean (SEM), #*p* < 0.001 (versus controls), **p < 0.01, *p < 0.05. *Scale bar* = 100 μm

MOG-inoculated animals developed a typical chronic MOG-EAE pattern (Fig. 1A) with MMS = 3.76 ± 0.28, AUC = 85.89 ± 11.73, dDO = 14.50 ± 0.47, and d50 score = 2.06 ± 0.44. Percentage of demyelination of the spinal cord was found 22.20 ± 1.89 % and 14.80 ± 1.34 % (*p* < 0.01) during the acute and chronic phases, respectively (Fig. 1B). Control animals (CFA inoculated animals) did not develop EAE.

Tissue sections from acute and chronic phases were compared to control animals. Hematoxylin staining revealed perivascular infiltrates (cells/mm^2^) within the white matter of the spinal cord in the acute phase (123.90 ± 9.89 cells/mm^2^) that was reduced during the chronic phase (87.80 ± 6.85 cells/mm^2^; *p* < 0.01) (Fig. 1C, F1–H2). Axonal loss assessed by Bielschowsky staining was found semi-quantitatively more extensive in the chronic phase than the acute (*p* < 0.05) and control group (*p* < 0.001), respectively (Fig. 1D, F1–H2). Microglial/macrophagic populations were increased in the acute phase (101.40 ± 4.63 cells/mm^2^ from control animal levels 10.44 ± 2.45 cells/mm^2^; *p* < 0.001) and then decreased towards the chronic phase (83.67 ± 5.77 cells/mm^2^; *p* < 0.05 versus acute and *p* < 0.001 versus controls) (Fig. 1E, I1–K2).

### Pattern of NgR expression inside and peripherally to EAE lesions of the spinal cord

In the spinal cord of controls, the highest NgR signal was obtained from neurons of the gray matter (>80 % of the total signal) and the rest from axonal elements (DAPI-negative structures).

**Fig. 2:**
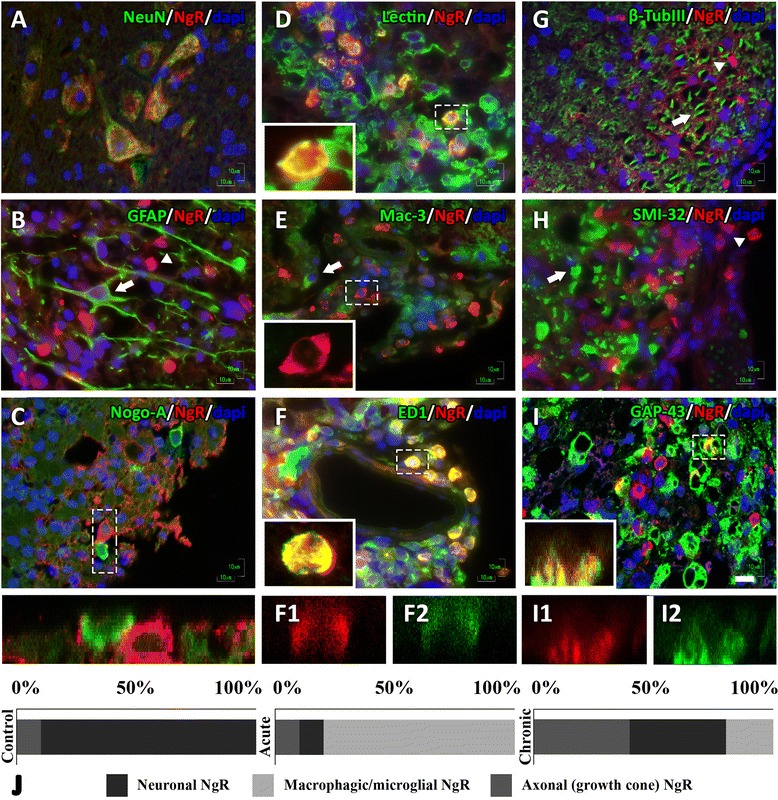
Cellular and axonal localization of the NgR protein in the acute phase of EAE. Neuronal expression (**A**) was acquired from the motor neurons of the gray matter, while the protein was absent from astrocytes (**B**) and oligodendrocytes (**C**) of the adjacent lesioned white matter. The macrophagical expression was restricted to subpopulations of microglia/macrophages as shown by the lectin staining (**D**) in activated ED1-positive macrophages and absent from Mac-3-positive macrophages (**E**, **F**). Axonal NgR was even more restricted, and while it was absent from the main axonal tract (**G**) or damaged sites, positive for non-phosphorylated form of neurofilament SMI-32 (**H**), it was detected at regenerative GAP-43^+^ axonal growth cones (**I**). *Inserts* **C**, **F1**, **F2**, **I1**, and **I2** represent z-stack scans 20 steps (0.3 μm) made in sections of 6 μm. **C** shows the contact point of two cells while **F1**, **F2**, **I1**, and **I2** coexpression is shown on two different channels of the respective pseudocolor. *Arrowheads* indicate the NgR^+^ cells, and *arrows* indicate the negative NgR structures. **J** NgR % levels and its subcellular façade in controls, acute phase, and chronic phase of EAE. *Scale* = 10 μm

In the acute phase, neuronal NgR signal (Fig. 2A) was decreased compared to controls (15.85 ± 9.09 % NeuN^+^NgR^+^ double-positive cells from 82.37 ± 7.81 % of controls; *p* < 0.001), was absent from astrocytes and oligodendrocytes (Fig. 2B, C), and found to be highly expressed in the lesioned white matter (306.7 ± 32.06 cells/mm^2^, 75.6 ± 9.04 % lectin^+^NgR^+^ inflammatory cell double positive) (Fig. 2D). NgR was found to be expressed in specific microglial/macrophagic cells; 49.6 ± 9.5 % were ED1^+^ (Fig. 2F), but Mac-3 negative (Fig. 2E), while the remaining percentage (~10 % the total signal) was found in axonal structures and specifically only in regenerative growth cones (GAP-43^+^ structures, Fig. 2I) and nowhere else throughout the axon (β-TubIII and SMI-32 negative, Fig. 2G, H). On the contrary, this specific localization was not observed in control animals.

In the chronic phase, the inflammatory process declines with a diminished appearance of inflammatory cells resulting in a decreased detection of cellular NgR localization within residual inflammations (<20 % of the total signal) while increasing the axonal localization in growth cones (IntDen, 195.975 ± 33.931) alongside a similar increase, 2.5-fold (39.44 ± 8.61 cells/mm^2^ versus acute 15.85 ± 9.09 cells/mm^2^; *p* < 0.01) in neurons of the gray matter of the spinal cord. NgR localization in each phase is shown graphically in Fig. 2J.

### Analysis of LINGO-1, p75, and TROY mRNA expression in the spinal cord by real-time PCR

**Fig. 3:**
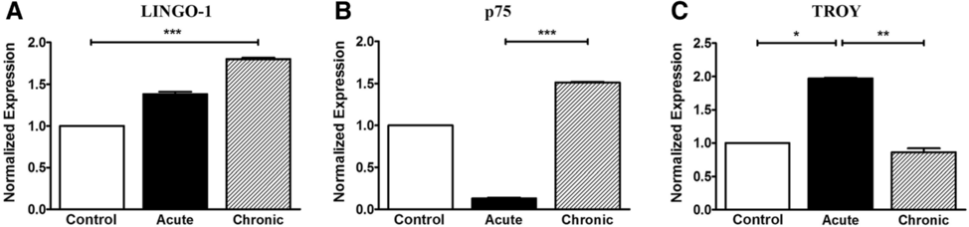
mRNA levels of coreceptors LINGO-1, p75, and TROY in the spinal cord of EAE animals. Levels of mRNA expression in the spinal cord by real-time PCR analysis for the coreceptors (**A**) LINGO-1, (**B**) p75, and (**C**) TROY, respectively. *Error bars* indicate the standard statistical error of the mean (SEM), ***p < 0.001, **p < 0.01, *p < 0.05

The messenger RNA (mRNA) levels of the NgR coreceptors were found to fluctuate, depending on the stage studied. Expression of LINGO-1 in the spinal cord was increased in the acute phase compared to controls and followed a statistically significant increase in the chronic phase (*p* < 0.001 compared to controls) (Fig. 3A). The corresponding normalized expression of p75 showed a decrease during the acute phase but increased significantly in the chronic phase (*p* < 0.001 compared to the acute phase) (Fig. 3B). On the contrary, TROY levels in the spinal cord appeared elevated only in the acute phase (*p* < 0.05 compared to controls and *p* < 0.01 compared to the chronic phase) and returned to the levels of controls in the chronic phase (Fig. 3C). Results were verified by the quality control, GAPDH analysis (Additional file 1: Figure S1).

### LINGO-1, p75, and TROY expression and cellular localization in the spinal cord

**Fig. 4:**
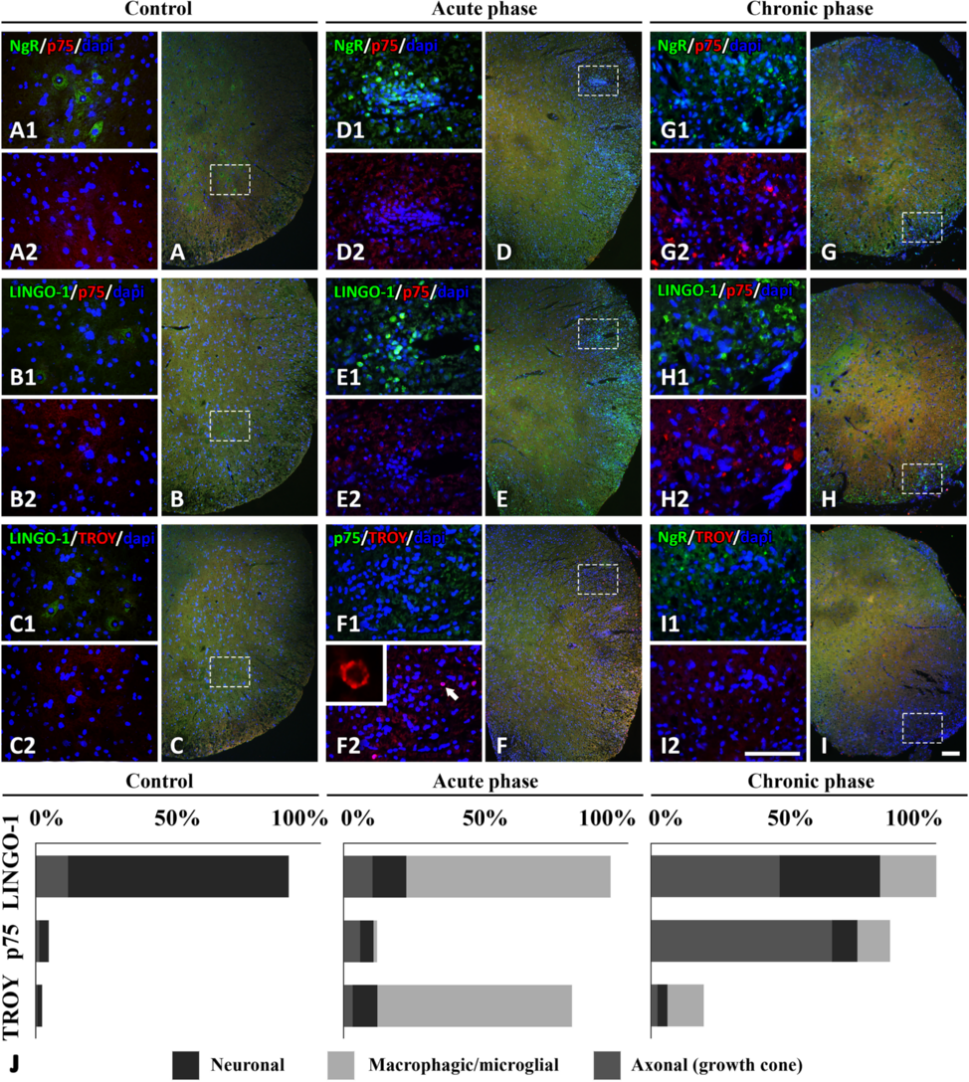
Protein expression of NgR and coreceptors (NgR complex) in the different phases of EAE. Double-fluorescent images indicating the expression of NgR, LINGO-1, p75, and TROY in serial spinal cord sections in controls (**A**–**C**), in the acute phase (**D**–**F**), and the chronic phase (**G**–**I**), respectively. *Inserts* represent magnified areas (*dashed rectangles*) where the highest expression of the molecules was found. **A1**–**C2** shows the neuronal expression of NgR and LINGO-1 to the motor neurons of the gray matter, and there is negligible expression of p75 and TROY. **D1**–**F2** shows the macrophage expression of NgR, LINGO-1, and TROY around the inflammatory infiltrates of the white matter, and there is the complete absence of p75. **G1**–**I2** shows the axonal expression of NgR, LINGO-1, and p75, which was present in DAPI-negative structures, while there is negligible expression of TROY in the residual inflammatory infiltrates. The *arrow* in **F2** represents enlarged TROY^+^ cell insert. **J** LINGO-1, p75, and TROY % levels with their subcellular façade in different phases of EAE. *Scale* = 100 μm

The protein expression of the NgR coreceptors exhibited a temporal profile in accordance with their respective mRNA expression levels. In controls, LINGO-1 was mostly expressed in motor neuronal bodies of the spinal cord while coreceptors p75 and TROY were almost negligible both in the gray and white matter of the spinal cord (Fig. 4A–C). Their expression fluctuated, like that of NgR, mainly within the inflammatory foci (Fig. 4D), depending on the phase studied.

The expression of LINGO-1 was increased in the perivascular inflammatory foci during the chronic phase (467.8 ± 48.18 cells/mm^2^; Fig. 4E1) while a similar increase was detected in axonal structures (IntDen, 249.156 ± 26.177; Fig. 4H1). LINGO-1 was found to be colocalized with both NgR (90.25 ± 5.36 % coexpression) and TROY (87.80 ± 7.15 % coexpression) in the acute phase while a corresponding colocalization was found between NgR (88.91 ± 7.50 % coexpression) and p75 (86.32 ± 9.29 % coexpression) in the chronic phase. Double immunofluorescence also revealed that the majority (91.42 ± 4.88 %) of ED1^+^ cells coexpressed LINGO-1 in the acute phase and an equal, high ratio of GAP-43^+^ axonal structures (90.71 ± 5.63 %) coexpressed LINGO-1 in the chronic phase.

Expression of p75 was increased only in the residual inflammatory foci of the chronic phase (IntDen 121.521 ± 15.709, versus acute 15.375 ± 1.399 and controls 2.650 ± 0.245; *p* < 0.001 for all comparisons) to axonal structures (Fig. 4G2, H2, D2, E2, A2, B2). On the other hand, expression of TROY was restricted within inflammatory cells at the lesion sites of the acute phase (106.7 ± 9.57 cells/mm^2^ versus chronic 15.56 ± 3.76 cells/mm^2^ and controls 1.48 ± 0.34; *p* < 0.001 for all comparisons) (Fig. 4F2, I2, C2). Coreceptor % levels in each phase are shown graphically in Fig. 4J. Taken together, these data suggest LINGO-1 and TROY as mainly acute-phase responders, expressed by the macrophages/microglia, while p75 is complementarily expressed in growth cones during the chronic phase.

### NgR expression in correlation with axonal regeneration and pathology

**Fig. 5:**
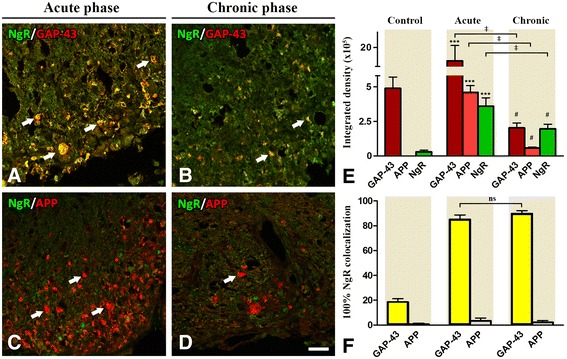
NgR protein expression in correlation with axonal regeneration and degeneration markers. Double-fluorescent immunostaining of NgR with molecular markers GAP-43 (**A**, **B**) and APP (**C**, **D**), respectively. **E** The data is displayed as integrated density (in arbitrary units) GAP-43, APP, and NgR in controls, acute phase, and chronic phase. **F** Percentage of GAP-43^+^ or APP^+^ axons coexpressing NgR. *** and # denote *p* < 0.001 for comparison of the acute and chronic phases, respectively, compared to controls, ‡*p* < 0.001 for comparisons indicated on the bars. *ns* non significant comparison. *Scale* = 20 μm

The increase of GAP-43 in the lesioned areas of the acute phase coincides widely with the increase of NgR signal (IntDen 350.255 ± 60.395) (Fig. 5A, E), in contrast to the adjacent normal-appearing white matter and compared to controls (29.731 ± 1.878; *p* < 0,001). These data are in accordance with our previously published data for Nogo-A [23]. The coexpression rate was up to 85.09 ± 3.57 %. The same pattern was observed in the chronic phase to a lesser extent since the two marker intensities exhibited an equivalent twofold decrease (195.975 ± 33.931) (Fig. 5B, E). The coexpression rate was also high and equal to 89.82 ± 2.3 %. In contrast, NgR failed to be found colocalized with APP, neither in the acute nor in the chronic phase of the disease (Fig. 5C, D, F).

### NgR macrophagic/microglial phenotype

**Fig. 6:**
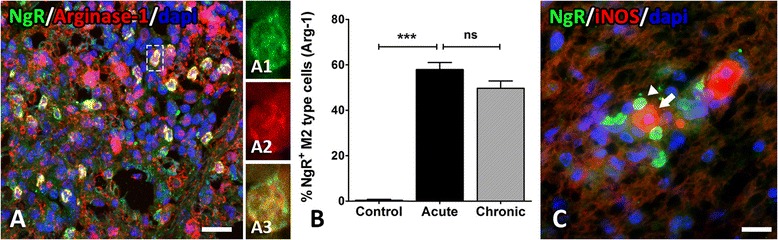
M1–M2 phenotype of NgR^+^ macrophagic/microglial populations within the inflammatory foci of the spinal cord. **A** A high expression of the Arginase-1 marker was observed in NgR^+^ cells (juxtapositions of separate color channels in **A1**–**A3**) within the acute lesions in the white matter. **B** Levels did not differ significantly in the chronic phase. **C** No connection (coexpression) was observed for iNOS^+^ (*arrows*) and NgR^+^ (*arrowheads*) cells in both acute and chronic phases. ***p < 0.001, *ns* non significant comparison. *Scale* = 20 μm

The control group did not express any Arginase-1 or iNOS in the white matter of the spinal cord, inducers for M2 and M1 cell polarization, respectively. Arginase-1 was expressed twofold higher in the acute phase (51.33 ± 7.38 cells/mm^2^) compared to iNOS (21.11 ± 2.07 cells/mm^2^) and revealed a high degree of colocalization (86.03 ± 2.08 %) within NgR^+^ cells (Fig. 6A). NgR^+^ M2-type inflammatory cells were 57.94 ± 3.07 % within the inflammatory foci of the acute phase (Fig. 6B). On the contrary, iNOS^+^ macrophages/microglia did not coexpress NgR and were only found in close proximity to NgR^+^ cells (Fig. 6C). Levels of Arginase-1 and iNOS remained unchanged in the subsequent chronic phase. NgR^+^ M2-type cells were 49.68 ± 3.22 % within the residual inflammatory foci of the chronic phase (Fig. 6B) whereas an M1 ratio could not be calculated. These data suggest an interesting link between NgR receptor and the neurorestorative M2 phenotype of macrophages/microglia in EAE, further implying a possible role of this molecule in the repairing process.

## Discussion

The main finding of our present study is the phase-sensitive differential expression of the NgR complex in the neurodegenerative inflammatory environment of MOG-EAE. Our data indicate that NgR is expressed in motor neurons of the gray matter, regenerating axonal growth cones and the M2 microglial/macrophages. Surprisingly, we show for the first time that this expression of NgR on microglia/macrophages is selective for Arginase-1^+^ M2 microglia/macrophages. In agreement with the NgR localization, we showed that both mRNA and protein of coreceptor LINGO-1 exhibit the same pattern in the spinal cord of EAE animals. Furthermore, molecular and histological findings of p75 and TROY coreceptors revealed that their expression is complementary between acute and chronic phases, respectively.

The EAE course involves different phases of inflammation and axonal pathology in the spinal cord [27]. Previously, we established that the mRNA and protein levels of NgR along with its putative ligand, Nogo-A, are subjected to changes under this inflammatory demyelination of the CNS, showing a notable increase during the chronic phase of EAE [23]. Because of the high local heterogeneity of NgR expression, further studies were required in order to decode more refined actions within the spinal cord. In this study, we initially showed that NgR was predominately expressed in motor neurons of the spinal cord gray matter of control animals (>80 %). In vitro data have previously connected NgR’s neuronal presence to growth cone collapse following injury [1, 8, 30]. However, the spatial expression changed drastically during the main time points of EAE and seemed to shift in terms of cellular localization.

In the acute phase, we showed that the majority of the NgR protein signal (70–75 %) was detected in inflammatory cells of lesioned areas, while lesser signal was acquired from axonal elements of white matter (~10–15 %). Although B and T cell populations were not examined in this study, it has been previously published that NgR deficiency does not affect the immune cell repertoire [16] nor the clinical outcome of EAE [17]. On the other hand, NgR has been strongly connected to cell adhesion [31, 32], motility of microglia and its regulation [22, 33, 34]. Thus, specific markers were selected for each developmental stage of microglia/macrophages; lectin staining was applied for the identification of resting and activated microglia, Mac-3 to detect blood-borne macrophages, and CD68 (ED1) to label the phagocytic cells [6, 22]. Our data show that during the acute phase, NgR was highly expressed by ED1^+^ cells, revealing the connection of an outgrowth regulator to one of the key players of inflammatory regulation in EAE [35]. NgR’s microglial localization in microglia has also been studied in traumatic brain injury (TBI) [36], linking the molecule to developmental functions and aging.

During the chronic phase, the percentage of axonal localization increased to 40–45 % and neuronal to 30–40 % while the microglial/macrophage expression was dramatically reduced to <20 %. The latest reduction might be due to the less inflammatory burden of the phase, while the neuronal reappearance might be connected to axonal remodeling and plasticity of corticospinal tract found during EAE rehabilitation period [37]. Although we failed to detect NgR on normal (β-TubIII) or pathological (SMI-32) axonal tracts, a significant colocalization with GAP-43 was seen on regenerative axonal growth cones. Such result further strengthens our previous Nogo-A findings, establishing that interaction of Nogo-A and NgR in the different phases of the EAE course plays a significant role in disease progression, accounting for inhibition of neurite outgrowth [23].

Real-time PCR molecular analysis in conjunction with dIF tissue stainings of serial sections revealed the kinetics of NgR coreceptors. LINGO-1 followed NgR’s pattern motility, both level- and cell-wise. This suggests that these two molecules are regulated together and exhibit an activity-induced neural plasticity response, especially for the neuronal coexpression [38]. On the other hand, p75 and TROY exhibited a more specific phase-dependent expression. It has been reported that p75 is present in areas where no TROY exists and vice versa [39, 40], thus complementing their actions in order to activate the downstream pathway of NgR [41, 42]. We found that more than 25 % of macrophages of the acute phase were found to express TROY in their cytoplasm while no p75 was found. TROY protein has been found up-regulated in MS brain lesions [43]. The absence of p75 in TROY^+^ cells has been also observed in sciatic nerve (PNS) of rats after injury [22]. Conversely, in the chronic phase, the appearance of p75 in no DAPI-correlated structures appeared to be unaffected by the lack of TROY from residual macrophages. Furthermore, neither of two proteins appeared in controls, leading us to assume that this spatiotemporal rearrangement of the molecules at a systematic level is associated with the processes occurring in the various stages of EAE, seen under certain circumstances [42, 44, 45]. The significance of these findings would be increased by functional experiments (KO mice or blocking peptides for LINGO-1, p75, and TROY), which unfortunately are not feasible for the time being, establishing a weakness in this study.

In order to understand the possible role of NgR in the inflammatory processes, we correlated its expression with microglia/macrophage polarization [28, 46]. Our data showed that NgR is expressed only by M2 macrophages/microglia suggesting that it may have an important but unknown role so far for their functions [46, 47]. Previous studies on rat EAE lesions support a positive role of the inflammatory ED1^+^ macrophages for the promotion of the repair process and recovery [28]. Additionally, the role of M2 microglia has been proven beneficial in EAE, by creating an anti-inflammatory environment, accompanied by tissue repair [46]. As a de novo synthesis of NgR in macrophages is possible, as seen after sciatic nerve crush in rats [21, 22], it may further prevent the spread of inflammation in the adjacent normal-appearing white matter [22]. Taken together, along with the high ratio of ED1^+^ macrophages present within the inflammatory foci, we propose that those phagocytic Arginase-1^+^NgR^+^ cells contribute to inflammatory regulation facilitating the repair process in the tissue.

In conclusion, we provide descriptive evidence for a possible action of NgR within inflammatory lesions of EAE acute and chronic phases. We show that NgR, LINGO-1, and TROY are expressed by macrophages of the acute phase and that NgR is also expressed on GAP-43^+^ axonal growth cones. Interestingly, the majority of NgR^+^ macrophages present in the inflammatory foci acquire the anti-inflammatory M2 phenotype which might ultimately lead to area clearance. As the kinetics of NgR, LINGO-1, p75, and TROY are tightly regulated and interchange both cellularly and time-wise, we propose that this system might be involved in the regulation, resolution, and repairing local processes after the inflammatory axonal injury in the spinal cord of EAE animals.

## Conclusions

This study demonstrates the expression kinetics of the Nogo receptor complex in autoimmune demyelinating lesions of EAE. Our data supports a phase-driven differential expression of all the molecules of the complex with a distinct temporal profile pattern, thereby defined by the EAE course. We further provide a possible underlying mechanism based on the selective expression milieu of NgR in GAP-43^+^ axonal growth cones and its coexpression in Arginase-1^+^, M2 phenotype alternatively activated macrophages.

## Additional file

Additional file 1: Figure S1. Bielschowsky staining, immunofluorescence, and real-time PCR quality controls. (A1–4) Typical images of scores 0–3 in Bielschowsky silver staining. (B, C) Preabsorption assay for LINGO-1 and TROY in serial sections of chronic and acute phases, respectively (the phase where signal is most abundant). (B1) LINGO-1 antibody specifications: rabbit polyclonal, Abcam ab23631, LOT N/A, dilution 1:300. (B2) LINGO-1 peptide specifications: rabbit, Abcam ab25890, LOT #GR41007-1, incubation with ab in 10× molecular ratio. (C1) TROY antibody specifications: goat polyclonal, Santa Cruz sc-13711 (E-19), LOT #H0707, epitope mapping near the C-terminus of TROY of mouse origin, dilution 1:100. (C2) TROY peptide specifications: goat, Santa Cruz sc-13711 P, LOT #B0402, incubation with ab in 10× molecular ratio. (D, E) Antibody specificity test for p75 and NgR with the use of another antibody (different company) recognizing a different epitope in serial sections of chronic and acute phases, respectively (the phase where signal is most abundant). (D1) p75 antibody #1 specifications: mouse monoclonal, Abcam ab8877, LOT GR136825-1, ME20.4, dilution 1:400. (D2) p75 antibody #2 specifications: mouse monoclonal, Santa Cruz p75 (B-1) sc-271708, LOT #J0611, epitope mapping between amino acids 393–427 at the C-terminus of NGFR p75 of human origin, 1:100. (E1) NgR antibody #1 specifications: rabbit polyclonal, Santa Cruz sc-25659 (H-120), LOT E1209, epitope corresponding to amino acids 31–150 mapping near the N-terminus of Nogo-R of human origin, dilution 1:100. (E2) NgR antibody #2 specifications: rabbit polyclonal, Abcam ab26291, LOT N/A, epitope from within residues 150–250 of rat Nogo receptor, dilution 1:100. (F) β-actin real-time PCR quality control showing the specific amplification products on agarose gel and the melting curves of their respective genes; curve identifier: light green TROY, orange p75, dark green LINGO-1. (G) mRNA levels of coreceptors LINGO-1, p75, and TROY in the spinal cord of EAE animals by real-time PCR analysis using GAPDH as a second, quality control, house-keeping gene. The levels of mRNA expression for the coreceptors (G1) LINGO-1, (G2) p75, and (G3) TROY, followed the same pattern with those that underwent β-actin analysis. Error bars indicate the standard statistical error of the mean (SEM), ****p* < 0.001, ***p* < 0.01. Black scale bar = 20 μm.

## Abbreviations

ANOVA: Analysis of variance
APP: β-Amyloid precursor protein
CFA: Complete Freund’s adjuvant
CNS: Central nervous system
DAPI: 4′,6-Diamidino-2-phenylindole
EAE: Experimental autoimmune encephalomyelitis
GFAP: Glial fibrillary acidic protein
GPI: Glycosylphosphatidylinositol
Iba-1: Ionized calcium-binding adaptor molecule-1
iNOS: Inducible nitric oxide synthase
LFB: Luxol fast blue
LRR: Leucine-rich-repeat
MOG: Myelin oligodendrocyte glycoprotein
MS: Multiple sclerosis
NeuN: Neuronal nuclei
SEM: Standard error of the mean
