# Supplementary figures and images for: Nogo receptor complex expression dynamics in the inflammatory foci of central nervous system experimental autoimmune demyelination
Source: J Neuroinflammation. 2016 Oct 11;13:265. doi: 10.1186/s12974-016-0730-4 (PMC5057208; doi:10.1186/s12974-016-0730-4)

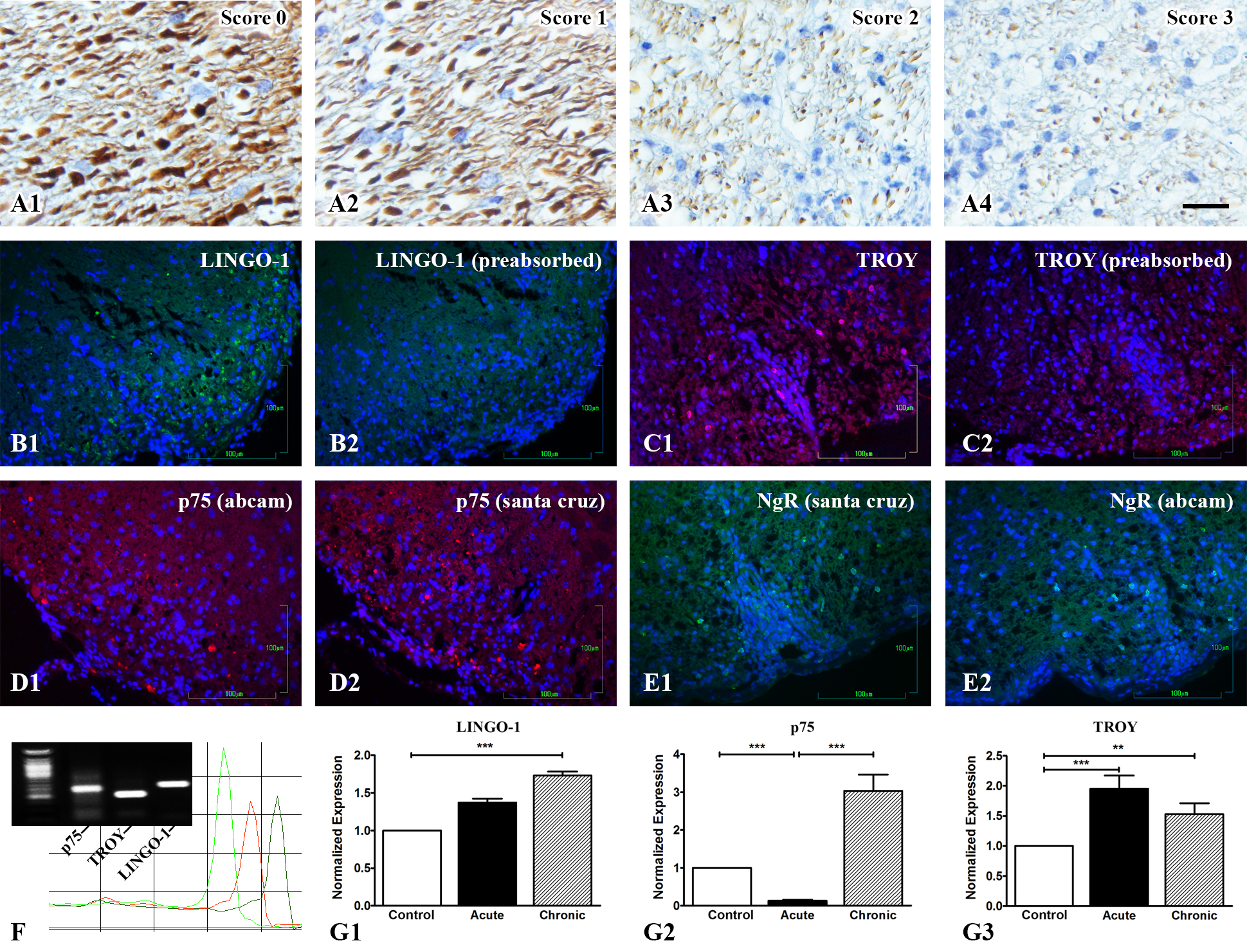

Supplement: Additional file 1: Figure S1. — Bielschowsky staining, immunofluorescence, and real-time PCR quality controls. (A1–4) Typical images of scores 0–3 in Bielschowsky silver staining. (B, C) Preabsorption assay for LINGO-1 and TROY in serial sections of chronic and acute phases, respectively (the phase where signal is most abundant). (B1) LINGO-1 antibody specifications: rabbit polyclonal, Abcam ab23631, LOT N/A, dilution 1:300. (B2) LINGO-1 peptide specifications: rabbit, Abcam ab25890, LOT #GR41007-1, incubation with ab in 10× molecular ratio. (C1) TROY antibody specifications: goat polyclonal, Santa Cruz sc-13711 (E-19), LOT #H0707, epitope mapping near the C-terminus of TROY of mouse origin, dilution 1:100. (C2) TROY peptide specifications: goat, Santa Cruz sc-13711 P, LOT #B0402, incubation with ab in 10× molecular ratio. (D, E) Antibody specificity test for p75 and NgR with the use of another antibody (different company) recognizing a different epitope in serial sections of chronic and acute phases, respectively (the phase where signal is most abundant). (D1) p75 antibody #1 specifications: mouse monoclonal, Abcam ab8877, LOT GR136825-1, ME20.4, dilution 1:400. (D2) p75 antibody #2 specifications: mouse monoclonal, Santa Cruz p75 (B-1) sc-271708, LOT #J0611, epitope mapping between amino acids 393–427 at the C-terminus of NGFR p75 of human origin, 1:100. (E1) NgR antibody #1 specifications: rabbit polyclonal, Santa Cruz sc-25659 (H-120), LOT E1209, epitope corresponding to amino acids 31–150 mapping near the N-terminus of Nogo-R of human origin, dilution 1:100. (E2) NgR antibody #2 specifications: rabbit polyclonal, Abcam ab26291, LOT N/A, epitope from within residues 150–250 of rat Nogo receptor, dilution 1:100. (F) β-actin real-time PCR quality control showing the specific amplification products on agarose gel and the melting curves of their respective genes; curve identifier: light green TROY, orange p75, dark green LINGO-1. (G) mRNA levels of coreceptors LINGO-1, p75, and TROY in the spinal [file 12974_2016_730_MOESM1_ESM.jpg]
